# Clinician Attitudes and Perceptions of Point-of-Care Information Resources and Their Integration Into Electronic Health Records: Qualitative Interview Study

**DOI:** 10.2196/60191

**Published:** 2025-05-26

**Authors:** Marlika Marceau, Sevan Dulgarian, Jacob Cambre, Pamela M Garabedian, Mary G Amato, Diane L Seger, Lynn A Volk, Gretchen Purcell Jackson, David W Bates, Ronen Rozenblum, Ania Syrowatka

**Affiliations:** 1 Clinical and Quality Analysis Mass General Brigham Somerville, MA United States; 2 Division of General Internal Medicine Brigham and Women's Hospital Boston, MA United States; 3 Massachusetts College of Pharmacy and Health Sciences (MCPHS) Boston, MA United States; 4 Vanderbilt University Medical Center Nashville, TN United States; 5 Intuitive Surgical Sunnyvale, CA United States; 6 Harvard Medical School Boston, MA United States; 7 Harvard TH Chan School of Public Health Boston, MA United States

**Keywords:** point-of-care tools, clinical decision support, artificial intelligence, point-of-care information, online databases, efficiency, quality of care, electronic health records, health information system interoperability, machine learning

## Abstract

**Background:**

Electronic health records (EHRs) are widely used in health care systems across the United States to help clinicians access patient medical histories in one central location. As medical knowledge expands, clinicians are increasingly using evidence-based point-of-care information (POCI) resources to facilitate clinical decision-making in medical practices. While these tools can improve clinical outcomes, few studies have assessed clinicians’ opinions on integrating them with EHRs.

**Objective:**

This study aims to assess clinicians’ attitudes and the perceived value of POCI resources for finding medication- and disease-related information in clinical practice and their integration with EHRs.

**Methods:**

Semistructured interviews were conducted with 10 clinicians from various roles and specialties between December 2021 and January 2022 at Brigham and Women’s Hospital in Boston, Massachusetts. A content analysis approach was used to examine participants’ responses and feedback on their current use of POCI resources, barriers and facilitators, mobile app use, and recommendations for improved integration.

**Results:**

Of the 10 participants, 6 (60%) were female, 9 (90%) were aged <40 years, and 8 (80%) had ≤10 years of experience in clinical practice. While UpToDate was the most preferred disease-related information resource (n=9, 90%), preferences for medication-related resources varied, with 2 (20%) participants favoring Micromedex, 2 (20%) favoring Lexicomp, 2 (20%) favoring Brigham and Women’s Hospital–specific drug administration guidelines, 2 (20%) favoring UpToDate, and 1 (10%) favoring Medscape. Most participants used their preferred tools weekly. Most clinicians preferred comprehensive POCI tools with clear, navigable layouts that eased and quickened the search for information. Features such as heavy text density, the lack of citations, and frequent log-ins to access the tool were viewed as barriers that limited content legibility, credibility, and accessibility. Access-related, tool-specific, and integration-related barriers were reported to negatively impact clinical workflow. Most (n=8, 80%) of the participants reported currently using mobile apps, reasoning that they facilitated quick and convenient searches for information; however, frequent updates, time-consuming log-ins, and high text density on smaller screens posed challenges. Most participants favored further integration of POCI resources with EHRs, with all reporting them being currently available as embedded links that launch externally. Some recommended that further integration would allow us to leverage existing POCI tool features, such as chatbots and knowledge links, as well as aspects of artificial intelligence and machine learning, such as predictive algorithms and personalized alert systems, to enhance EHR functionality.

**Conclusions:**

Participants favored integration to improve usability and optimize workplace efficiency by reducing the amount of time spent seeking answers to their medication- and disease-related questions. Recommendations on integration highlighted the need for stakeholder input in developing clinical decision support tools and interfaces that leverage advancements in artificial intelligence and machine learning while not compromising user experience or increasing time spent on tasks.

## Introduction

### Background

Being a practicing clinician requires navigating and retaining an immense and evolving breadth of knowledge as the field of medicine progresses and standards of care change. To address the knowledge gaps that arise during a busy workday, clinicians use a variety of methods to support their clinical decision-making [1]. To be effective, clinical resources must be accurate, reliable, helpful, and quickly accessible in addressing clinicians’ questions on various medical topics [1-3].

While it is common to use search engines, such as Google, clinicians have increasingly come to rely on evidence-based online information resources, such as point-of-care information (POCI) tools, to help answer medication- and disease-related questions that arise during patient care [1]. POCI tools are evidence-based online information websites and databases that support clinical decision-making by providing quick and reliable responses to clinical questions [1,4]. Clinicians can use these types of clinical decision support systems to find peer-reviewed literature and clinical practice guidelines to inform standards and practices used during patient care delivery [1,4]. BMJ Best Practice, DynaMed, Micromedex, and UpToDate are some examples of POCI tools that have different content areas of focus with a wide range of features, layouts, and modes of access [1,4]. Health care organizations often offer access to a variety of online resources via institutional subscriptions, including collections of electronic textbooks and journals, search tools for bibliographies and abstracts of primary literature (eg, PubMed or Embase), and more focused resources, such as the Natural Medicines Comprehensive Database [4].

Online information resources often include barriers that challenge their users at the point of care. For example, clinicians have reported that some POCI tools are difficult to navigate, thereby prolonging their search time, lack depth in their explanations, and provide unsatisfactory answers, all of which might affect their acceptance and use [2,5]. However, these resources have been proven to enhance the delivery of care and clinical outcomes when properly implemented by improving diagnostic accuracy and treatment plans [6,7]. Therefore, a need exists for innovative methods to address these barriers and optimize POCI tools to best meet clinical needs.

There have been increasing efforts to integrate POCI tools with electronic health records (EHRs) to improve usability and clinical workflow [8]. Examples include the ability to access a resource directly from the EHR without having to launch it via an external browser and the option to download apps to access the tools via mobile devices. If effectively implemented, integrating these tools may help optimize the speed of information retrieval and reduce clinician burden during searches for information to inform clinical practice [8]. Furthermore, clinicians’ input on the integration of these tools can help identify potential areas for improvement due to their experience with using these tools in the clinical setting [2,5,8].

### Objectives

This study aimed to assess clinicians’ attitudes, current use, and the perceived value of POCI tools for finding medication- and disease-related information. In addition, this study examined the attitudes of clinicians toward the integration of POCI tools with EHRs, identified barriers and facilitators, and solicited recommendations for improved integration.

## Methods

### Study Design and Setting

We conducted semistructured interviews with clinicians (30-45 min) employed at the Brigham and Women’s Hospital (BWH) in Boston, Massachusetts, from December 2021 to January 2022.

### Study Participants, Sampling, and Recruitment

Our targeted recruitment method sought to engage clinicians from a diverse range of specialties and experiences. Physicians, physician assistants (PAs), pharmacists, and registered nurses providing patient care in internal medicine, infectious diseases, neurology, and critical care were included. We used purposive sampling to recruit from a pool of participants who completed a usability study on the combined DynaMed and Micromedex with Watson tool (DynaMedex, hereafter referred to as the combined tool), given their experience with this POCI tool [2,9,10]. Participants involved in the usability study were asked if they would be interested in taking part in a subsequent study; thereafter, all interested individuals were invited to participate in this study. The combined tool was selected for evaluation as it represented typical medication- and disease-related information resources that clinicians commonly use. It also incorporated artificial intelligence (AI)–powered features, such as a conversational interface to facilitate search.

### Interview Guide

A semistructured interview guide was developed in collaboration with subject matter experts (MGA, DLS, and DWB), a human factors expert (PMG), and an experienced qualitative researcher with expertise in the topic investigated in the study (RR; Multimedia Appendix 1). We added preliminary questions to better understand participants’ clinical backgrounds and their previous use of the POCI resources to gather feedback on their experiences using these resources in their particular practice. The guide was then piloted with 1 clinician and the internal research team to identify areas for improvement and ensure perspectives could be shared without input from the interviewer. We integrated the feedback received from the pilot by revising the guide to address gaps and concerns.

The revised and final guide contained 15 open-ended questions divided into 4 parts. Part 1 comprised questions about participants’ demographics and clinical backgrounds. Part 2 addressed their use of current clinical tools, part 3 inquired about Micromedex and DynaMed use, and part 4 explored their experiences with the combined tool and recommendations for integrating it with the EHR. The combined tool was deployed as a use case to assess participants’ perceptions of the value of integrating the information resources.

### Ethical Considerations

The study was exempted by the Mass General Brigham Institutional Review Board (protocol #2021P001813). All participants provided verbal informed consent to be interviewed and recorded before starting the interview. Participants could opt out and stop the interview at any time without providing a reason. All interview data were deidentified to protect participants’ privacy and confidentiality and stored on password-protected computers behind the firewall at Mass General Brigham. Each participant received a US $50 Amazon gift card as compensation for their time participating in the study.

### Data Collection and Analysis

All interviews were conducted on Zoom (Zoom Communications, Inc) by SD. The video recordings were transcribed by a professional transcription company. Using a content analysis approach, 2 reviewers (SD and MM) independently abstracted data from the transcripts and entered it into a Microsoft Excel document organized according to the sections of the interview guide. Similar ideas were grouped together to identify and summarize a set of concepts for each interview topic [11]. The reviewers cross validated each other’s groupings to ensure that the data analysis was consistent and replicable [12]. To further validate their findings and ensure transparency in the review process, the reviewers met with the experienced researchers (RR and AS) to discuss discrepancies and achieve consensus [12]. The results were structured according to the concepts derived from each section of the interview guide and were titled as follows: participant demographics and clinical backgrounds, overview of current practices, use of preferred web-based tools in current practice, barriers to using web-based information resources in current practice, preferred mobile apps, and attitudes on integrating web-based information resources into the EHR. We subsequently report the study findings using the terms *a few* (30% or fewer participants), *some* (>30% and <70% of the participants), or *most* (>70% of the participants) to describe the frequency of perspectives shared and specific patterns in participant responses. Data saturation was reached after 10 interviews, as no new significant themes emerged, with key themes consistently repeated across participants, indicating that further interviews would not provide substantially different insights [13].

## Results

### Participant Demographics and Clinical Backgrounds

We contacted 12 clinicians from BWH and interviewed 10 with diverse clinical backgrounds and experiences (Table 1). Of these 10 participants, 3 (30%) physicians, 3 (30%) pharmacists, 2 (20%) PAs, and 2 (20%) nurses were interviewed. Most clinicians identified as female (n=6, 60%), were aged <40 years (n=9, 90%), and had ≤10 years of experience practicing (n=8, 80%). Half (5/10, 50%) of the participants self-reported being in leadership roles in their practice, and there were equal numbers working in inpatient and outpatient settings.

**Table 1:** Demographic characteristics and clinical backgrounds of study participants (N=10).

Characteristics		Participants, n (%)
**Sex**		
	Female	6 (60)
	Male	4 (40)
**Age group (y)**		
	21-30	2 (20)
	31-40	7 (70)
	41-50	1 (10)
**Clinical role**		
	Physician	3 (30)
	Physician assistant	2 (20)
	Pharmacist	3 (30)
	Registered nurse	2 (20)
**Specialty**		
	Critical care	3 (30)
	Infectious diseases	1 (10)
	Internal medicine	4 (40)
	Neurology	2 (20)
**Duration of practice (y)**		
	<1-5	3 (30)
	6-10	5 (50)
	11-20	1 (10)
	21-30	1 (10)
**Duration of practice at BWH** ^a^ **(y)**		
	<1	1 (10)
	2-5	5 (50)
	6-10	4 (40)
**Clinical setting**		
	Inpatient	5 (50)
	Outpatient	5 (50)
**Leadership role**		
	Yes	5 (50)
	No	5 (50)

^a^BWH: Brigham and Women’s Hospital.

### Overview of Current Practices

All interviewed clinicians reported using various web-based resources to look up information in clinical settings (Table 2). There was substantial overlap in the resources used to find medication- and disease-related information. Participants favored web-based tools that were available within the EHR as links from a pull-down resource menu. Most participants used POCI tools, such as Micromedex (6/10, 60%), UpToDate (5/10, 50%), or Lexicomp (3/10, 30%), to find medication-related information. UpToDate (9/10, 90%) or primary literature databases (4/10, 40%), such as PubMed and Google Scholar, were used most often to find disease-related information. A few participants reported accessing web-based content resources, such as hospital-based policies or clinical practice guidelines, before using POCI tools because they provided more specialty-specific information. A few participants relied on direct communication with others, such as calling a pharmacy or consulting with colleagues for medication-related questions.

**Table 2:** Reported use of web-based medication–related and disease-related information resources by study participants (N=10)^a^.

Name of resource	Brief description^b^	Type of resource	Participants who reported using the medication-related resource, n (%)	Participants who reported using the disease-related resource, n (%)
Clinical practice guidelines^c^	Clinical practice guidelines provide best practice recommendations based on evidence-based research on how to identify and treat medical conditions [14].	Content resource	2 (20)	2 (20)
DynaMed	DynaMed is a clinical decision support tool that provides clinicians with evidence-based information for quick answers at the point of care [15].	POCI^d^ tool	N/A^e^	1 (10)
Hospital-based policies^f^	Hospital-based policies are the rules and guidelines adopted by hospitals that guide decisions and standards of practice [16].	Content resource	1 (10)	1 (10)
Lexicomp (through UpToDate)	Lexicomp is a drug reference database that provides evidence-based recommendations to help clinicians treat and counsel patients on their conditions and medical histories [17].	POCI tool	3 (30)	N/A
Medscape	Medscape is an online platform that provides access to medical news, expert feedback, and medical and disease information while supporting clinicians with professional education opportunities, including continuing medical education [18].	POCI tool	1 (10)	1 (10)
Micromedex	Micromedex is a comprehensive database that provides information on medications, diseases, and toxicology to support diagnostic and treatment decision-making in clinical practice [19].	POCI tool	6 (60)	N/A
Primary literature databases^g^	Primary literature databases are platforms that provide access to journal articles on health and medical sciences and other related content [20].	Content resource	2 (20)	4 (40)
UpToDate	UpToDate is a subscription-based clinical decision support resource that helps clinicians access evidence-based medical information while facilitating patient care [21].	POCI tool	5 (50)	9 (90)

^a^Percentages added to >100% because participants reported using multiple tools.

^b^The descriptions were taken from the official websites of the products and tools.

^c^Examples are Johns Hopkins Antibiotic Guide, Fast Facts, drug administration guidelines, and the Centers for Disease Control and Prevention Opioid Guideline.

^d^POCI: point-of-care information.

^e^N/A: not applicable.

^f^Examples are Brigham and Women’s Hospital policies, such as the empiric antibiotic guide.

^g^Examples are PubMed, Embase, and Google Scholar.

### Use of Preferred Web-Based Tools in Current Practice

#### Overview

All participants expressed a preference for specific web-based resources. A few participants preferred Micromedex (2/10, 20%) or Lexicomp (2/10, 20%) to find medication-related information, and 10% (1/10) of the participants used both tools interchangeably. Other preferred medication resources included clinical practice guidelines (ie, BWH-specific drug administration guidelines; 2/10, 20%), UpToDate (2/10, 20%), and Medscape (1/10, 10%). UpToDate (9/10, 90%) was the most favored resource for finding disease-related information. Moreover, 10% (1/10) of the participants preferred using Medscape.

#### Reasons for Selection

The reasons for selecting preferred tools were similar for medication- and disease-related resources. Most participants preferred their tool due to its targeted information, function, and capabilities, while some favored their tool for its comprehensiveness in combining both disease- and medication-related information into one place.

Most participants found it convenient to use tools that they were familiar with and were quickly accessible, often through the EHR. For example, one physician stated the following:

*[I will] probably go to UpToDate because it is easily accessible, and I [am] familiar with that. UpToDate is my go-to...because of the familiarity and ease of access.* [Internist]

Most participants also acknowledged that content and layout influenced the selection of their preferred tool, especially when the tool’s design made it easy to find information quickly:

[It is] designed with the clinician in mind in the sense that you can search through it, you can click by topic, you can click within subtopics...You can narrow in on what it is that you need help with.

Neurologist

Given their reliance on evidence-based information, most participants selected tools based on their assessments of whether the information provided was reliable and credible. For instance, some participants favored evidence-based disease-related resources because they helped them learn about unfamiliar topics and clinically reason through possibilities while generating differential diagnoses for a medical case:

It’s a good start for me to go to something like UpToDate for a disease state that I’m not familiar with, just because it does give you a general recommendation, but it also ties in a lot of the most recent guidelines of literature.

Critical care pharmacist

Finally, a few participants were encouraged to use their tool because it offered continuing medical education (CME) credits:

Because it also provides CME, I want to make sure that I have my information passed through because I can collect some CME through my searches.

Internist

#### Experience With Preferred Tool

Most participants reported positive experiences using their preferred information resource, finding it useful, usable, and informative. Some participants expressed their appreciation for clear layouts, bulleted formats, in-depth explanations, and embedded links to supporting literature, which helped provide evidence-based recommendations:

I think both are...usable. I appreciate that Micromedex has both the short version of things, but then you can also look at an in-depth version that expands beyond the monograph and to citing more data and the reason behind certain recommendations.

Infectious disease pharmacist

I think [it is] comprehensive. I think...[it is] accurate. I like that [there are] links to references so you can choose to read the primary research.

Neurologist

One participant felt it was important that their tool provided details on when the information was updated, and another mentioned the positives of being able to access information that is specific to their institution’s policies through their tool on the EHR system.

While most participants reported positive experiences using their information resources, they also expressed shortcomings, such as a lack of depth in certain areas and longer times spent looking for information. These caveats are further explained as follows:

Sometimes [there are] specific questions that are a little bit more in depth that [are not] really covered by some of these resources, but [that is] expected...Resources [cannot] cover everything.

Critical care pharmacist

I think [in] UpToDate...you need to scroll a little bit more and the way [it is] laid out, I find it just takes me longer to read it.

Neurologist

In addition, a recurrent theme mentioned was high text density and large paragraphs, which made navigation difficult, especially when trying to quickly skim through the text. Some participants did not feel that the navigation bars helped to reduce the time spent looking for information, as mentioned in the following statements:

What I think [it is] missing is...sometimes the paragraph format is too long...I find that [it is] a little bit too much information. Sometimes, when [you are] reading something new or when [you are] not sure what [you are] looking for, [it is] harder to zone into exactly the point that you want.

Neurologist

It was also noted that it was more time consuming to find specific answers regarding disease-related questions as opposed to medication-related ones because disease-based sections were more text dense. For example, one participant stated the following:

You pretty much know, no matter what medicine you look at, how much content, and how is it laid out, and [that is] very predictable. I think for diseases, it can be a little bit more vast. Sometimes, you might need to narrow your search a little bit more or figure out which section you want to jump to because you really do not have time to read the whole thing, nor are the sections always finite because [they] can sometimes be super-long depending on the disease.

Internist

#### Accessing Resources

Most participants accessed their preferred resource through their institutional EHR system. Once in the EHR, some participants described a variety of pathways to their preferred resource, which all involved clicking an embedded link to launch an external web browser that redirected to the resource website. Information resources could be accessed through the resources drop-down in the EHR, the KnowledgeLink portal of the EHR, or the institution’s handbook containing electronic references for clinical use. Resources were also located in the hospital employee intranet through Citrix Workspace. These pathways were discussed in the following statement:

For at least me, specifically, within Epic [there are] tabs that they have embedded links for clicking on access to UpToDate, Micromedex, so [that is] the easiest way for me to do so.

Critical care pharmacist

A few participants accessed the resources directly through a web browser because they found this to be a quicker and easier method. They expressed dislike for Internet Explorer (Microsoft Corporation) as a default browser, stating that it is not user-friendly. One pharmacist explained as follows:

I honestly generally just pull up a web browser tab and do it that way, because [that is] through Chrome, which is much quicker than if you click on it through Epic. It goes through Internet Explorer, which is an awful browser.

Infectious disease pharmacist

#### Frequency of Use

Half (5/10, 50%) of the participants estimated using their preferred medication-related resource either once or a couple of times a week. Conversely, 30% (3/10) of the clinicians used it daily, and a few used it monthly, depending on their clinical needs. Regarding preferred tools for disease-related queries, 60% (6/10) of the participants used them on a weekly basis, 30% (3/10) used them monthly, and 10% (1/10) used them on a daily basis.

#### Timing of Use

Most participants gave a broad range of answers for when they used their preferred resource in the context of patient care, regardless of their clinical background. All participants accessed their tool at different times throughout the patient care journey. Approximately 70% (7/10) of the clinicians used their tool before a patient encounter, often to learn more about the patient’s condition; 70% (7/10) of the clinicians used the resources during encounters, and 60% (6/10) of the clinicians used them after an encounter while writing notes or looking up additional information. Moreover, 60% (3/5) of the inpatient clinicians also reported using their preferred resource during rounds. Most participants said they would be most likely to use a combination of the aforementioned time points, depending on their needs, as noted in the following response:

If I [have not] heard of a disease, then I would obviously want to learn about it just before going to see the patient...so I can understand what [we are] doing a little bit better if it impacts why [they are] in the hospital. Then, maybe after, if they have the new diagnosis on the differential, I would look into it just to make sure I totally understand what [they are] thinking is going on for a patient.

Critical care nurse

#### Page Utility

Most participants were on a specific page of the EHR when they decided to seek medication- or disease-related information in their tool. For instance, most participants reported using the clinical notes page when looking for disease-related information from their tool, while the medications list page was most frequently used when looking for medication-related guidance in their tool, as described in the following statements:

[There are] medication searches and disease searches, and those definitely have different views. The medication searches would happen on the medication tab, versus disease searches would probably be more likely in the Notes or the Past Medical History section.

Infectious disease pharmacist

I would most likely be on the medication administration page just because that [is] where the medication order is, and I have that in front of me. [That is] what I need to access to look up the information.

Internal medicine nurse

Notably, one pharmacist mentioned being on the results page of the EHR when they launched their medication-related resource, as articulated in the following statement:

If I’m looking to see about an adverse event, I might be on the Results tab to see how labs are trending compared to what might be published.

Infectious disease pharmacist

Another pharmacist reported using their tool for disease-related questions while navigating the medications list page to better understand how medications may impact a patient’s condition.

### Barriers to Using Web-Based Information Resources in Current Practice

Most participants reported a range of access-related, tool-specific, and integration-related barriers that negatively impacted clinical workflow (Multimedia Appendix 2). Specifically, some users found it challenging to rely on institutional permissions to access resources, efficiently navigate to their preferred tools from the EHR, and find information in the resources when the pages were too wordy.

### Preferred Mobile Apps

Details about the use of preferred mobile apps and barriers to use in current practice are described subsequently. The use of mobile apps to access POCI resources reported by the study participants is given in the subsequent sections.

In total, 8 (80%) of the 10 participants reported currently using mobile apps to access POCI resources (Table 3). These participants accessed these apps on phones or tablets when a desktop computer was not available:

Folks that are running around and seeing dozens and dozens of patients, and only might have phone access between seeing patients.

Infectious disease pharmacist

**Table 3:** The current use of mobile apps reported by study participants to access point-of-care information resources.

Characteristic			Participants, n (%)^a^		Clinical roles
**Name of mobile app (n=8)**					
	Lexicomp	1 (12)		1 pharmacist	
	Medscape	1 (12)		1 nurse	
	Micromedex	2 (25)		1 MD^b^ and 1 pharmacist	
	UpToDate	6 (75)		2 MDs, 1 pharmacist, and 2 PAs^c^	
	Other^d^	6 (75)		2 MDs, 2 pharmacists, 1 PA, and 1 nurse	
**Frequency of use (n=8)**					
	Daily	3 (38)		2 PAs and 1 nurse	
	Once a week	1 (12)		1 pharmacist	
	Once a month	2 (25)		1 MD and 1 pharmacist	
	A few times per year	2 (25)		2 MDs	
**At what point in the patient encounter were the apps used? (n=8)**					
	Before	6 (75)		3 MDs, 1 PA, 1 pharmacist, and 1 nurse	
	During	3 (38)		1 PA and 2 pharmacists	
	After	6 (75)		3 MDs, 1 PA, 1 pharmacist, and 1 nurse	
	Other (pages, off shift, etc)	1 (13)		1 pharmacist	
Patient rounds (inpatient clinicians only; n=5)			1 (20)		1 MD

^a^Centers for Disease Control Opioid Guideline, Fast Facts, Johns Hopkins Antimicrobial Guide, Sanford Guide, University of California–San Francisco Outpatient Medicine Handbook.

^b^MD: physician.

^c^PA: physician assistant.

^d^Frequency reflects the number of participants who mentioned using these mobile apps.

Most participants used UpToDate, Micromedex, and specialty-specific mobile apps that were also available as web-based versions. Most of the participants cited the ease of access, enhanced portability, the speed of information retrieval, and familiarity with the resources as reasons for using these apps. They found that these mobile apps were a convenient way to pull up specific resources quickly, with 38% (3/8) of the participants using these tools daily (Table 3). For example, one PA expressed the following:

UpToDate is certainly the thing I’m the most comfortable [with]. I think especially when [you are using a mobile device, I feel like your ability to navigate it quickly is a key to success actually, depending on what you’re looking for. Also, when I’m looking something up on my phone [it is] not like [I am] looking to spend a long time reading something of interest. [It is] usually like, “I need to know something right now.”

Internal medicine PA

Some participants used mobile apps to avoid barriers related to using desktop tools:

On my phone, I can just click on the app. I [do not] have to worry about logging in and [it is] faster. Going to the computer, you have to log in, load and all that stuff...I only use the app.

Internal medicine nurse

One physician appreciated that the mobile versions of the tools promised the same standards as the web versions. Most clinicians used the apps before and after patient encounters, and of the 5 inpatient clinicians, only 1 (20%) used an app during rounds. Physicians avoided using the tool during patient encounters, considering it would be inappropriate to be on a mobile phone in front of patients.

Barriers to mobile app use included challenges navigating copious amounts of text on smaller screens, updates, and log-ins. Some participants reported needing the content to be made app friendly:

[There is] usually either lots of text, if [you are] talking about disease state management, or lots of information, if [you are] talking about a drug monograph...you do a lot of scrolling on your phone to find what you need. It takes a little bit longer, versus on the larger screen.

Infectious disease pharmacist

One pharmacist described finding the log-in process time consuming:

Some of the barriers, like I mentioned, any Apple application having updates or login maybe if [they are] retyping a login and stuff like that that either cost time, or...[if] it was a hospital code [and] I forgot it [then] I [would not] have access.

Critical care pharmacist

Participants who no longer used the apps cited various barriers that affected their user experience. One physician reported no longer using a mobile app because the subscription renewal process and logging in were challenging and time consuming.

### Attitudes on Integrating Web-Based Information Resources Into the EHR

#### Current Status of Integration With EHR

All participants reported that their preferred web-based resources were not fully integrated but were generally available as embedded links that launched external web pages:

[It is] a completely separate link. [It is] not like if you have atrial fibrillation in your note and [there is] a link [to] UpToDate. No, you have to open it as a resource.

Neurologist

A few participants described partial integration for medication orders, such as direct hyperlinks to medication information through an external browser:

In certain medication orders, there are hyperlinks directly so that it goes straight to the medication [information] from order in Epic.

Infectious disease pharmacist

One participant commented on the customizability of the EHR, noting that they were able to ease their access to their preferred tool by personalizing it to meet their needs:

*You can set your settings. I had an Epic person...set that up for me. Epic modified my toolbar in [the] Epic menu and rearranged things...It tells you what you use the most. There [are] different ways to arrange things the way you want.* [Neurology PA]

Participants considered full integration to provide quick and direct access to medication- and disease-related information within the EHR:

I honestly think the less there is clicking and the more something is going to directly take you to what you want the more it’s going to get used.

Internal medicine PA

#### Preferences Regarding Integration

Most respondents favored accessing the resources via embedded links to external pages:

Probably better if it launches separately because when in [the] EHR, you [do not] want [what is] in front of your face to be hijacked by your search, necessarily.

Internist

A few preferred the information to be presented within the EHR. They reasoned that this would provide faster access to information by avoiding browser-related interruptions. For example, 20% (2/10) of the participants stated the following:

If it can be further integrated into EHR, it would definitely be useful. I think everybody benefits from getting quicker information.

Internal medicine nurse

I think in EHR. The only reason I say that is because of consistent browser issues.

Neurology PA

#### Suggestions for Improving Integration

Participants proposed a variety of solutions to improve integration with the EHR. To facilitate the quick retrieval of information resources, a couple of participants suggested the following:

You need to have it kind of like in a panel approach, so like, our EHR, Epic, has a side bar and then the main page, and the only way I can see it working well is if it was somehow integrated into that side bar, or in some sort of pop-up that you could still [see] the chart behind it.

Infectious disease pharmacist

You could even put it under the chart search bar so that [you are] really specifying are you searching the chart or are you searching information. You could also integrate it into the search so when you search the chart, [it is] like,“‘[Here is] the patient information’ or ‘[Here is] information about it.’ What are you searching?” I think that it might be cool to put it where alerts pop up so if the contraindications pop-up comes up, it could have a link [to the resource].

Neurologist

[It would] be nice if there was either a dedicated activity within the EHR or a window or a corner or an icon where, when you click into it, it expands, and then [you will] see [the information resource], and then a search box opens up. Then, when you click enter, then [it is] okay if it opens up a new browser or window. Up to that point, it would feel a lot more integrated because you [are] able to type in your search string while [you are] still within your EHR.

Internist

It was also important to maintain a simple and user-friendly display with direct pathways to the resources embedded throughout the EHR, especially to make it easier for users to find the most relevant resource for their questions, as described in the following statement:

If I have a question and am not sure which part of the software I should be using, that is problematic, so there should be a way to guide you to the most appropriate application based off [the] question. The pathway needs to be really streamlined—it has to be really clear this is where I need to go.

Internist

Some participants identified existing features of the EHR that could be leveraged for integrating resources, given their current usefulness. For example, one participant discussed the potential use of KnowledgeLinks:

I can see a world in which you put diverticulitis down as a visit diagnosis and there [is] a little ⓘ next to diverticulitis and you click it, and it takes you to that page. That would be sweet for me.

Internal medicine PA

A chatbot was another feature favored by a few clinicians that could assist in finding information:

You know the little chat box at some websites, like [you are] on [a] travel [website] and [it] is like, “I can help?” Maybe if it was there and it was either just available as a search bar or if it had little suggestions based on what [you are] typing or what page [you are] on or stuff like that.

Neurologist

Some suggestions for integration involved using currently evolving capabilities to optimize the usability of the EHR. A few participants noted that AI could be used to identify critical information from patient charts, such as potential contraindications based on the medication list. They added that AI features could even evolve to provide alerts based on information from the resource for specific patient charts. This is expressed in the following comment:

You could incorporate [AI] to alert to contraindications. We get a bunch of alerts like, “Your patient is due for vaccines” or “Your patient needs the flu vaccine.” I work [in] the inpatient [setting], but primary care, they also get a lot of alerts about guidelines around screening, stuff like that.

Neurologist

#### Concerns About Integration

Despite the widespread support for integration, some participants expressed reservations. They argued that integration must center on functionality, with a few citing concerns that it could interrupt their workflow by altering the current view of the EHR. For example, one participant stated the following:

A little bit of me is like if this is going to mess up how Epic looks, I’d rather have it be an external page that pins because then [it is] not in any way altering your view of [the] Epic screen.

Internal medicine PA

Similarly, most participants preferred that their resources existed as embedded, externally launched links, emphasizing that a poorly designed integration could lead to launching windows that obscure their view of patient information:

[It is] nice for me to be able to control whether or not I want to move that pop-up window or browser to the side or if I want to spend a moment and read all of it. This is where it sounds a little bit like [it is] paradoxical in a way. I want to integrate it, but I [do not] necessarily need it to take over the screen that [I am] working at.

Internist

The link right now that we have for UpToDate opens up a clunky Internet Explorer browser within the EHR, and [it is] not that user-friendly overall. I [do not] suggest that. Then, also, if it opens up in a new tab in your EHR in the first place, that kind of negates the point because then you [cannot] see patient [information] at the same time because [it is] covering it up.

Infectious diseases pharmacist

## Discussion

### Principal Findings

As medical knowledge rapidly evolves, web-based POCI resources have become common tools used to support complex decision-making in clinical practice. Although these tools have the potential to improve workplace efficiency, diagnostic accuracy, and treatment plans, few studies have examined clinician attitudes toward integrating them with EHRs [6,7]. This qualitative study examines clinician attitudes, current use, and perceived utility of POCI tools for answering medication- and disease-related questions during patient care delivery. Attitudes, barriers, and facilities regarding the integration of POCI tools with EHRs were also evaluated, in addition to recommendations for enhancing integration. Most participants preferred POCI tools and mobile apps with clear layouts that allowed for quick and easy access to medication- and disease-related information. Most participants supported the integration of POCI tools with EHRs, while some provided recommendations to improve the usefulness of the EHR, which included the addition of existing POCI tool features, such as chatbots and knowledge links, as well as AI and machine learning functions, such as predictive algorithms.

We conducted semistructured interviews with various clinicians to understand the current uses of POCI tools, barriers to and facilitators of use, and recommendations for integrating these tools with EHRs. We found that there were a variety of POCI tools used, most of which were large information databases that provided medication- or disease-related information, such as UpToDate and Micromedex, allowing users to access a wide breadth and depth of answers in a central location. In addition to an appreciation for comprehensiveness, there was a strong preference for POCI tools that eased the search for evidence-based information via clear, user-friendly layouts consisting of features such as bullets and embedded links that directed users to relevant information. The absence of such layouts in existing tools made it challenging for most users, who found disease-related pages to be text heavy, rendering navigation difficult and prolonging their search for information.

### Comparison to Prior Work

These reported barriers and facilitators are consistent with previous studies where clinicians favored tools with minimalistic designs because they valued finding information easily and quickly [2,22]. The participants reasoned that complex layouts that are either too text dense or require multiple steps to locate relevant information reduced content visibility and tool accessibility, respectively [2,22]. They also emphasized that it was important for tools to cite sources supporting clinical information because it improved clinicians’ understanding of the evidence for standards of care, which can enable adherence to clinical guidelines [6,22].

Participants also reported positive experiences using mobile web apps of their preferred POCI resources, reasoning that their portability, accessibility, and speed allowed them to find information quickly. In addition to frequent log-ins, participants reported that high levels of text density were still a barrier they experienced while using these apps. A previous study reported similar findings, recommending that improvements to text and content clarity as well as offline access can support better participant experiences using mobile web app versions of POCI resources [23].

Regarding integration, participants had positive attitudes toward integrating these tools into the EHR, with most preferring that they be launched through embedded links while a few favored that they display directly within the EHR. It was important to most clinicians that integration leverage features that exist in both POCI tools and the EHR, including chatbots, knowledge links, and search functions, to improve resource navigability and usability. Recent studies reported positive experiences with integrating tool features into the EHR, including automatic calculations and assistance in treatment planning that improved clinician workflow and heightened quality care delivery [7,24].

Given the recent focus on AI and machine learning in medicine, a few participants proposed the use of AI predictive features to alert clinicians to relevant medical information, such as medication contraindications and patients in need of more individualized care. In recent studies, AI, natural language processing, and machine learning algorithms were found to increase the accuracy of clinical decision support systems in areas ranging from identifying at-risk patients requiring regular ophthalmology visits to facilitating laboratory diagnostics that account for patients’ medical histories [25-27]. If implemented, some studies emphasized that alert systems apply customizable alert filtration schemes that tailor to the specific, clinically relevant needs of the clinician to limit the number of low-value alerts that contribute to alert fatigue [6-8,28,29]. These predictive features may also reduce clinicians’ cognitive load and increase clinical task efficiency by reducing time spent searching for medically relevant information [27]. Effectively implementing predictive features could help detect clinicians’ latent needs and improve patient safety through increases in diagnostic accuracy [30,31]. This will undoubtedly play an important role in the future. Overall, improvements to POCI resources and their integration with the EHR may have positive downstream effects on task efficiency by decreasing the number of actions performed to reach a desired outcome, resulting in enhancements to the clinician work experience by reducing time spent seeking information and increasing time delivering quality care [8,32].

### Limitations

First, this study was limited to a small sample of clinicians with previous experience using POCI resources at the same academic medical institution. However, we were able to recruit a range of clinician types with a variety of experiences in the clinical setting to provide perspectives on the use of POCI resources and their integration with EHRs. Second, the insights and recommendations provided may not be generalizable to all clinical settings and clinicians’ experiences, which may differ according to several factors, including the availability of health IT, geographic location, access to resources, and clinical experience. It is important that future studies account for a wide range of clinicians’ perspectives on updates to POCI resources and EHRs to identify opportunities for improvement and workflow considerations that benefit clinicians’ experiences in clinical settings and their ability to effectively care for their patients. Future research must also be conducted across a wider range of clinical settings and geographic locations to account for differences in access to and experience with health IT.

### Conclusions

We evaluated the preferred features and uses of POCI resources of clinicians as well as their attitudes toward integration with the EHR. Clinicians had strong preferences for POCI resources that were comprehensive, user-friendly, and allowed them quick access to medication- and disease-related information. Suggestions for integration included optimizing EHR usability to improve workflow efficiency by reducing time spent navigating resources and searching for relevant clinical information. Novel advancements in AI and machine learning could also be incorporated into the EHR to assist clinicians with identifying risk factors, including adverse events, which could impact patient health outcomes. This study demonstrated that including clinician input in the development of clinical decision support and health IT is key to ensuring that clinical workflow needs can be met to improve both the clinician and patient experience in medical settings.

## Appendix

Multimedia Appendix 1 Semistructured interview guide.

Multimedia Appendix 2 Barriers to using web-based information resources in current practice with frequencies (number of participants who mentioned these barriers) and examples reported by study participants (N=10).

## Abbreviations

AI: artificial intelligence
BWH: Brigham and Women’s Hospital
CME: continuing medical education
EHR: electronic health record
PA: physician assistant
POCI: point-of-care information

## Data Availability

The datasets generated or analyzed during this study are available from the corresponding authors on reasonable request.
