# Supplementary material for: Clinician Attitudes and Perceptions of Point-of-Care Information Resources and Their Integration Into Electronic Health Records: Qualitative Interview Study
Source: JMIR Med Inform. 2025 May 26;13:e60191. doi: 10.2196/60191 (PMC12149773; doi:10.2196/60191)
Supplement: Multimedia Appendix 1 [file medinform_v13i1e60191_app1.docx]

**MULTIMEDIA APPENDIX 1. Semi-structured Interview Guide**

**Part 1: Background Questions**

1. What is your clinical role (any specialty)?
2. Approximately how long have you been practicing?
3. How long have you been practicing at BWH?
4. Do you have a leadership position?
   1. If yes, what is your role?
5. Gender?
6. Age?

**Part 2: Current Practices**

In this part, we will discuss your current practices regarding finding drug and disease information.

1. What do you do when you have a question about medications and/or diseases?
   1. What resources(s) do you use to find the current best practice regarding **medications**?
   2. What resources(s) do you use to find the current best practice regarding **diseases**?
2. Which information resource do you use the most to find **evidence-based information about medications**? Why?
   1. What are your experiences using the information resource? (Do you find it useful?)
   2. What are your barriers to using the information resource?
   3. How do you access the information resource?
      1. Is the information resource integrated within your EHR? If yes, please elaborate.
   4. When do you use this information resource?
      1. Before, During, After patient encounter
         1. At what point of care do you use the information resource most often?
      2. What page of the EHR are you on most often when using the information resource?
   5. How often do you use the information resource?
      1. Daily, Once a week, Once a month, A few times per year
3. Which information resource do you use the most to find **evidence-based information about diseases**? Why?
4. What are your experiences using the information resource? (Do you find it useful?)
5. What are your barriers to using the information resource?
6. How do you access the information resource?
   1. Is the information resource integrated within your EHR? If yes, please elaborate.
7. When do you use this information resource?
   1. Before, During, After patient encounter
      - At what point of care do you use the information resource most often?
   2. What page(s) of the EHR are you on most often when using the information resource?
8. How often do you use the information resource?
   1. Daily, Once a week, Once a month, A few times per year
9. Do you use mobile applications (e.g., an app downloaded from the app store on your phone) to find drug/disease relevant information? If yes:
10. Which application(s)?
11. Why do you use this application(s)?
12. Is a web version of the tool available? If yes,
    1. What prompts you to use the mobile app rather than the web version of the tool?
13. How often do you use the application(s)?
    1. Daily, Once a week, Once a month, A few times per year
14. What are your experiences using this application(s)?
    1. Do you see value in using this application(s)
    2. What are your barriers to using the application(s)?
15. When do you use this application(s)?
    1. Before, During, After patient encounter
       - At what point of care do you use the application(s) most often?
    2. What page(s) of the EHR are you on most often when using the application(s)?
16. Is the application(s) integrated within your EHR? Do you find it helpful?

**Part 3: Familiarity with DynaMed and Micromedex**

Intro: In this part we will discuss your familiarity and usage of DynaMed and Micromedex.

1. Are you familiar with DynaMed?

| If yes | If no |
| --- | --- |

| Ok, we’ll move on to the next question (question #6) |
| --- |

Do you use it?

| Yes | No |
| --- | --- |

Why do you not use it?

1. Why do you use it?
2. How often do you use it?
   1. Daily, Once a week, Once a month, A few times per year
3. What are your experiences using DynaMed?
   1. Do you see value in using DynaMed? Why?
   2. What are your barriers to using DynaMed?
4. When do you use DynaMed?
   1. Before, During, After patient encounter
      - At what point of care do you use DynaMed most often?
   2. What page(s) of the EHR are you on most often when using DynaMed?
5. Is DynaMed integrated within your EHR? If yes, do you find it helpful?
6. Are you familiar with Micromedex?

| If yes | If no |
| --- | --- |

| Ok, we’ll move on to the next question (question #6) |
| --- |

Do you use it?

| Yes | No |
| --- | --- |

Why do you not use it?

1. Why do you use it?
2. How often do you use it?
   1. Daily, Once a week, Once a month, A few times per year
3. What are your experiences using Micromedex?
   1. Do you see value in using Micromedex? Why?
   2. What are your barriers to using Micromedex?
4. When do you use Micromedex?
   1. Before, During, After patient encounter
      - At what point of care do you use Micromedex most often?
   2. What page(s) of the EHR are you on most often when using Micromedex?
5. Is Micromedex integrated within your EHR? If yes, do you find it helpful?

**Part 4: DynaMedex Integration into EHR.**

Intro: In this part, we will discuss your experiences with DynaMedex and your opinions about its integration into an EHR.

8) What are your experiences with using DynaMedex? Tell me about your experience.

9) In general, what are your thoughts about DynaMedex?

10) Do you see value in using DynaMedex? Why?

11) Do you think that DynaMedex should be integrated within the electronic health record?

1. If yes:
   1. Why?
   2. Where in the EHR would you like to see this and be able to access it? Be specific.
      1. E.g., Medications page, Home page, etc.
   3. Should the information be presented within the EHR, or would you prefer using a link that takes you directly to the information you need in DynaMedex?
2. If no:
   1. Why not?
   2. How would you like to access it?
   3. Is there anything you can suggest that could be done to support having it integrated within the electronic health record?

12) How valuable do you think it is to integrate DynaMedex into an EHR?

13) If DynaMedex is integrated into the EHR, at what point of care would you use it?

- 1. Before, During, After patient encounter

14) Would you use the DynaMedex Mobile App? Please elaborate, why/ why not?

15) Is there anything else you would like to share with us regarding DynaMedex?

- Do you have any additional thoughts about integration of the tool within an EHR?
- Are there any aspects of your workflow or interaction with the EHR that we didn’t talk about but would be important for us to keep in mind?
