# Supplementary material for: Clinician Attitudes and Perceptions of Point-of-Care Information Resources and Their Integration Into Electronic Health Records: Qualitative Interview Study
Source: JMIR Med Inform. 2025 May 26;13:e60191. doi: 10.2196/60191 (PMC12149773; doi:10.2196/60191)
Supplement: Multimedia Appendix 2 [file medinform_v13i1e60191_app2.docx]

**Multimedia Appendix 2.** Barriers to Using Web-based Information Resources in Current Practice with Frequencies (Number of Participants Who Mentioned These Barriers) and Examples Reported by Study Participants (N=10)

| Barriers | | Frequency, n (%) | Examples of descriptive quotes |
| --- | --- | --- | --- |
| **Access-related barriers** | | | |
|  | Lack of institutional permissions and subscriptions: Users relied on institutional access and found the renewal process and institutional subscription requirements to be inconvenient and time consuming. | 4 (40) | - “I think the requirement for an active account and membership sometimes can be a barrier because it asks the user to refresh your information because we get institutional licensure [and] permission to use it. Sometimes, I want to look up something and [it has] been either a while or [it is] time for them to renew my institutional permissions, and I have to actually go through that before I find [the information].” [Internist] - “If you [do not] have an UpToDate account, you [cannot] access it. If you look something up, it will just give you basic [information] and...to continue, you need to log in or have an account...I think you have to pay for it or something like that, but I wish it was just a little bit more accessible or free for health care providers.” [Internal medicine nurse] |
|  | Difficulty accessing tools remotely: Users found it challenging to work remotely or be off-site, which required additional layers of password protection and the use of VPN^a^ and negatively impacted their workflow. | 2 (20) | - “It’s a little annoying, especially now that we are working remotely because one of the ways that that happens is it asks if [you are] on campus. Somehow, it knows your IP address, and [that is] how you validate it. [That is] a little bit challenging. I know how to get through that just because of the VPN process, but I can see that as having been a barrier.” [Internist] - “I think one thing that can sometimes be a little bit [of] a pain sometimes within Epic and integration of Micromedex: If you [are not] necessarily on the [institutional] network, it is an extra step to make sure...it links to the appropriate login account, like the hospital account.” [Critical care pharmacist] |
|  | Required frequent log-ins: Users found it inconvenient to have to log in every time they needed to access tools. | 2 (20) | - “[It is] a lot easier for me to just quickly search on my phone, so [it is] right there, rather than trying to find the right site, and determining whether or not I have to log in, and all that stuff.” [Critical care pharmacist] - “On my phone, I can just click on the app. I [do not] have to worry about logging in and [it is] faster. Going to the computer, you have to log in, load and all that stuff.” [Internal medicine nurse] |
| **Tool-related barriers** | | | |
|  | Too wordy: Users reported that page layouts of some tools were too text dense, which made it difficult to find information quickly in fast-paced clinical environments. | 4 (40) | - “I feel like those sites can be very wordy. [It is] hard when [you are] just trying to find a very specific answer to one of your questions...[That is] certainly less ideal if [you are] in the clinical setting. I feel like you rarely have the time to be digging on UpToDate for your answer.” [Critical care nurse] - “UpToDate has that a little bit where you can choose the section, but then I find that [it is] a little bit too much information. Sometimes when [you are] reading something new or when [you are] not sure what [you are] looking for, [it is] harder to zone into exactly the point that you want.” [Neurologist] |
|  | Insufficient information in some subject areas: Users found that information lacked detail in some areas. | 1 (10) | - “Sometimes [there are] specific questions that are a little bit more in depth that [are not] really covered by some of these resources.” [Critical care pharmacist] |
|  | Lack of transparency on when information was updated: Users found that some POCI^b^ tools did not consistently provide information about when content was updated. | 1 (10) | - “I think [it is] more common when [there is] something new. Otherwise, then, as you read through something, if [there is] nothing new, they still tend to cite the date of a study or results from research. I think the ability to see the date and how new or stale it is, is something that I do like. [It is] not something that I feel like I always have to know, but if [it is] there, [it is] reassuring to see, ‘Oh, okay. This was just updated fairly recently’.” [Internist] |
| **EHR^c^ integration–related barriers** | | | |
|  | Required too many clicks: Users found it tedious navigating from the EHR to the web page of the information resource. | 2 (20) | - “I think sometimes it gets a little clunky. [There is] a lot of clicks to get from Epic to the actual page of the medicine that I am looking for, and I have a preference for that being simpler. It certainly can get there. [It is] not hard. [It is] just time consuming.” [Internal medicine PA^d^] - “Within the EHR itself, [I am] just thinking if [there is] a way for it to be more straightforward. It takes multiple clicks to get through, which is kind of annoying and [it is] not straightforward.” [Internist] |
|  | Insufficient information in some areas: Users found that the EHR did not provide enough information to support clinicians who were delivering care in certain cases (eg, contraindications and drug administration sites). | 2 (20) | - “You’ll get a contraindication error on Epic if you order something [that is] contraindicated with something else the patient is already on, but there [is not] a good way to be like, ‘Oh, my patient [is] on warfarin and X, and I want to quickly think about that ahead of time.’” [Neurologist] - “I know for a fact if I go on the resources in [the] EHR, [I am] not going to find [whether it is safe or not to administer medication to a different bodily area]. I obviously go under the administration, and I look through all the resources that are available, but the administration just tells you if it needs to be diluted [and] how to dilute it. It tells you if [it is] a medication that needs to be administered not fast or over 15 minutes or 10 minutes or whatever, but sometimes I’m looking for the administration site.” [Internal medicine nurse] |
|  | A lack of integration led to frequent switching back and forth from the information resources to the EHR: Users reported navigating back and forth from the EHR to the information resource, which interrupted their workflow. | 2 (20) | - “It would be great if there was integration right into your regular day workflow, rather than opening a new tab, searching something, maybe not finding it right away. Searching for something else, then finding it and going from there. To have integration would be awesome.” [Infectious disease pharmacist] - “I think having more than one location—the problem is because Epic [has] a...very redundant layout. I think in order for [users] to use these resources efficiently, you have to be redundant in terms of where you can access it but accessing it in a way [that is] simpler. For example, having to click on a separate tab to get to it is painful as opposed to knowing that [I am] likely to reference it within a hyperlink in the area where [I am] working.” [Internist] |

^a^VPN: virtual private network.

^b^POCI: point-of-care information.

^c^EHR: electronic health record.

^d^PA: physician assistant.
